# *Ensifer canadensis* sp. nov. strain T173^T^ isolated from *Melilotus albus* (sweet clover) in Canada possesses recombinant plasmid pT173b harbouring symbiosis and type IV secretion system genes apparently acquired from *Ensifer medicae*

**DOI:** 10.3389/fmicb.2023.1195755

**Published:** 2023-06-14

**Authors:** Eden S. P. Bromfield, Sylvie Cloutier, Michael F. Hynes

**Affiliations:** ^1^Ottawa Research and Development Centre, Agriculture and Agri-Food Canada, Ottawa, ON, Canada; ^2^Department of Biological Sciences, University of Calgary, Calgary, AB, Canada

**Keywords:** *Ensifer canadensis* sp. nov. T173^T^, complete genome sequence, phylogenetics, symbiosis plasmid, horizontal gene transfer, type IV secretion system (T4SS), plasmid conjugation systems

## Abstract

A bacterial strain, designated T173^T^, was previously isolated from a root-nodule of a *Melilotus albus* plant growing in Canada and identified as a novel *Ensifer* lineage that shared a clade with the non-symbiotic species, *Ensifer adhaerens.* Strain T173^T^ was also previously found to harbour a symbiosis plasmid and to elicit root-nodules on *Medicago* and *Melilotus* species but not fix nitrogen. Here we present data for the genomic and taxonomic description of strain T173^T^. Phylogenetic analyses including the analysis of whole genome sequences and multiple locus sequence analysis (MLSA) of 53 concatenated ribosome protein subunit (*rps*) gene sequences confirmed placement of strain T173^T^ in a highly supported lineage distinct from named *Ensifer* species with *E. morelensis* Lc04^T^ as the closest relative. The highest digital DNA–DNA hybridization (dDDH) and average nucleotide identity (ANI) values of genome sequences of strain T173^T^ compared with closest relatives (35.7 and 87.9%, respectively) are well below the respective threshold values of 70% and 95–96% for bacterial species circumscription. The genome of strain T173^T^ has a size of 8,094,229 bp with a DNA G + C content of 61.0 mol%. Six replicons were detected: a chromosome (4,051,102 bp) and five plasmids harbouring plasmid replication and segregation (*repABC*) genes. These plasmids were also found to possess five apparent conjugation systems based on analysis of TraA (relaxase), TrbE/VirB4 (part of the Type IV secretion system (T4SS)) and TraG/VirD4 (coupling protein). Ribosomal RNA operons encoding 16S, 23S, and 5S rRNAs that are usually restricted to bacterial chromosomes were detected on plasmids pT173d and pT173e (946,878 and 1,913,930 bp, respectively) as well as on the chromosome of strain T173^T^. Moreover, plasmid pT173b (204,278 bp) was found to harbour T4SS and symbiosis genes, including nodulation (*nod, noe*, *nol*) and nitrogen fixation (*nif*, *fix*) genes that were apparently acquired from *E. medicae* by horizontal transfer. Data for morphological, physiological and symbiotic characteristics complement the sequence-based characterization of strain T173^T^. The data presented support the description of a new species for which the name *Ensifer canadensis* sp. nov. is proposed with strain T173^T^ (= LMG 32374^T^ = HAMBI 3766^T^) as the species type strain.

## Introduction

The genus *Ensifer* consists of diverse species of soil bacteria and includes bacterial predators and agriculturally important species capable of fixing nitrogen in symbiotic association with legume plants such as alfalfa (*Medicago sativa*). This bacterial genus is currently divided into two major clades based on phylogenetic analysis of multiple housekeeping (core) genes (Martens et al., 2008; Kumar et al., 2017; Fagorzi et al., 2020; Kuzmanović et al., 2022). One of these clades is represented by the non-symbiotic species, *Ensifer morelensis* (Wang et al., 2002) and the predatory species, *Ensifer adhaerens* (Casida, 1982), and the other clade by symbiotic nitrogen fixing species such as *Ensifer meliloti* (de Lajudie et al., 1994) and *Ensifer medicae* (Rome et al., 1996). Based on comprehensive genomic and phenotypic data, Kuzmanović et al. (2022) proposed the recognition of these two clades as separate genera named *Ensifer* and *Sinorhizobium*, respectively. However, this proposal was not adopted by the Judicial Commission of the International Committee on Systematics of Prokaryotes (Oren and Garrity, 2022) and the genus *Ensifer* (Casida, 1982) remains intact.

In previous studies (Bromfield et al., 2001, 2010), bacteria were isolated from root-nodules of alfalfa and sweet clover (*Melilotus albus*) plants grown at a Canadian field site without a history of cultivation. Characterization of these bacterial isolates by phylogenetic analysis of four house-keeping gene sequences (Bromfield et al., 2010) resulted in the identification of several lineages that were assigned to the genus *Ensifer*. One of these lineages, represented by a bacterial strain designated T173^T^, was found to be novel and closely related to the type strain of *E. morelensis*. Strain T173^T^ was also found to possess a plasmid harboring nodulation (*nod*) and nitrogen fixation (*nif*) genes and to be able to elicit nodules on the roots of several *Medicago* and *Melilotus* species but not fix nitrogen.

In the present study our purpose was to use genomic, phylogenetic and phenotypic analyses to further characterize and verify the taxonomic status of strain T173^T^. Based on the results presented here a novel bacterial species named *Ensifer canadensis* sp. nov. is proposed.

## Materials and methods

### Bacteria

Novel strain T173^T^ was isolated from a root-nodule of a *Melilotus albus* plant grown at a field site in Ottawa, Ontario, Canada that had no known history of agriculture as described by Bromfield et al. (2001). Reference strains used in phylogenetic and genomic analyses are listed in Supplementary Table S1; reference strains used in different phenotypic tests are listed in relevant Tables and Figures in the main text or Supplementary material section. Bacterial strains were routinely grown on yeast extract-mannitol (YEM) agar medium (Tang et al., 2012) or modified tryptone yeast-extract (TY) agar medium with the following composition (g/L): tryptone (Oxoid, United States), 0.5; yeast-extract (Oxoid, United States), 1.0; calcium chloride dihydrate, 0.1; Bacteriological agar no. 1 (Oxoid, United States), 15.0. Bacteria were maintained at −80°C in 20% w/v glycerol. Strain T173^T^ was deposited in the BCCM/LMG Bacteria Collection, University of Ghent, Belgium as LMG 32374^T^ and in the HAMBI Microbial Culture Collection, University of Helsinki, Finland as HAMBI 3766^T^.

### Phenotypic characterization

Assessment of Gram-stain reaction of bacteria was done using the KOH method of Buck (1982).

Multiple tests including carbon source utilization and chemical sensitivity assays were carried out using BIOLOG GEN III MicroPlates (Biolog, United States) according to manufacturer’s instructions.

For analysis of fatty acids, bacteria were grown for 2–4 days on TY agar medium (Beringer, 1974) at 28°C. Bacteria were harvested and fatty acids extracted as described by Sasser (1990). Fatty acid identification was carried out using the Sherlock Microbial Identification System (MIDI) version 6.0 and the RTSBA6 database.

Electron microscopy was used to investigate cell morphology employing bacteria grown in modified TY broth at 28°C. For transmission electron microscopy (model, H-7000; Hitachi), bacterial samples were adsorbed to formvar coated copper grids and negatively stained with 1% phosphotungstic acid (pH 7.0). For scanning electron microscopy (model, Hitachi SU7000 FESEM), bacteria were adsorbed to Poly-L-Lysine coated silicon wafers (Ted Pella Inc., CA, United States). Samples were fixed using 3% glutaraldehyde in 0.1 M sodium Cacodylate buffer pH 7.2, dehydrated in a graded ethanol series and critical point dried in an Autosamdri-931 critical point dryer (Tousimis Research Corp, MD, United States). Wafers were mounted on aluminum stubs coated with a 6.5 nm layer of platinum in an Emitech K550V sputter coater (EM Technologies Ltd., Kent, United Kingdom).

Tests of ability to “track” bacterial prey cells to detect potential bacterial predatory activity of strain T173^T^ were carried out using plates of 0.1x Heart Infusion (BD Difco, United States) agar medium supplemented with 0.1% glucose as detailed by Martin (2002). Plates were incubated at 28°C for 7 days and any spreading growth of predator strains over cells of the prey strain was recorded. Bacterial test strains were *Micrococcus luteus* JCM 1464^T^ (as prey), *Escherichia coli* (EZ competent cells, Qiagen, Netherlands) (resistant to predation; negative control), *Ensifer adhaerens* Casida A^T^ (as predator) and *E. morelensis* (as predator).

Tests of acid production by bacteria grown on YEM agar medium for 21 days at 28°C were carried out as described previously (Bromfield et al., 2010).

The ability of bacteria to grow in LB broth medium at 30^°^C was assessed using 96-well microplates as detailed by Fagorzi et al. (2020). Growth was estimated (over a 48 h period) on the basis of optical density (595 nm) by taking readings at hourly intervals using a FLUOstar OPTIMA plate reader (BMG LABTECH, Germany).

Plant tests of nodulation and nitrogen fixation using seedlings from surface sterilized seed were done using Leonard jar assemblies (Vincent, 1970) (three replicate jars, two plants per jar) as described previously (Bromfield et al., 2010).

### Analyses of partial gene sequences

Sequences of 16S rRNA, *atpD*, *glnII*, *gyrB*, *recA*, and *rpoB* housekeeping genes were extracted from genome sequences and used for phylogenetic analyses. Sequence accession numbers are shown in Supplementary Table S1. Alignment of 16S rRNA gene sequences was done using the fast, secondary-structure aware Infernal aligner implemented in the online Ribosomal Database Project version 11.5 (Cole et al., 2014). Sequences of protein-encoding housekeeping gene sequences (*atpD*, *glnII*, *gyrB*, *recA*, and *rpoB*) were aligned using MUSCLE (Edgar, 2004).

Best fit substitution models were selected using ModelTest-NG (Darriba et al., 2020) implemented in CIPRES Science Gateway version 3.3 (Miller et al., 2010). Bayesian phylogenetic analyses were performed using MrBayes version 3.2.1 with default priors (Altekar et al., 2004) as previously described (Yu et al., 2014). Maximum-likelihood (ML) phylogenetic analyses (Guindon et al., 2010) were carried out using 1,000 non-parametric bootstrap replications to assess support as described by Tang et al. (2012). Phylogenetic trees from Bayesian and ML analyses exhibited similar topologies and therefore only Bayesian trees are shown in this work.

### Genome analyses

Bacterial cells for genomic DNA preparation were grown on modified TY agar plates for 2 days at 28°C. Genomic DNA was extracted from bacterial cells (washed twice in sterile water) using the Promega Wizard SV Genomic DNA Purification System (Promega, United States). The genomic DNA was purified using the DNeasy PowerClean Pro Cleanup Kit (Qiagen, Netherlands) according to manufacturer’s instructions.

Complete genome sequencing of strain T173^T^ was carried out at the Genome Quebec Innovation Centre, Montreal, Canada, using the Pacific Biosciences (PacBio) Sequel single-molecule real-time (SMRT) platform (Ardui et al., 2018) as described previously (Nguyen et al., 2018). Assembly of sequence reads was done using SMRTLink (v.7.0.0.63985) software (Pacific Biosciences of California, Inc.) and circularization was carried out using Circlator v.1.5.5 (Hunt et al., 2015).

Methods for estimating overall genome relatedness such as digital DNA–DNA hybridization (dDDH) and average nucleotide identity (ANI) have replaced the outdated and error prone DNA–DNA hybridization (DDH) method for bacterial species delineation (Chun et al., 2018; Meier-Kolthoff and Göker, 2019; Meier-Kolthoff et al., 2022). We calculated dDDH values for strain T173^T^ and reference strains of the genus *Ensifer* using the suite of algorithms implemented in the web-based Type Strain Genome Server (TYGS) (Meier-Kolthoff et al., 2013; Meier-Kolthoff and Göker, 2019; Meier-Kolthoff et al., 2022). The established dDDH threshold of 70% was used to delineate species boundaries (Meier-Kolthoff and Göker, 2019). ANI values were estimated using the FastANI method (Jain et al., 2018) implemented in the K base web server (Arkin et al., 2018). The established ANI threshold of 95–96% was employed for species circumscription (Richter and Rosselló-Móra, 2009; Lee et al., 2016; Ciufo et al., 2018).

To investigate horizontal transfer of symbiosis and related genes to strain T173^T^, comparative analysis of the complete sequence of the symbiosis plasmid (pT173b; accession no., CP083372) with the symbiosis megaplasmid (pWSM1115_2; accession no. CP088111) of *E. medicae* strain WSM1115 (Reeve et al., 2014), was done using GenomeMatcher (Ohtsubo et al., 2008) and Geneious Prime 2023.0.4^1^ software.

The BV-BRC web-based platform (Olson et al., 2023) was used to search for antibiotic resistance genes in the genomes of strain T173^T^ and reference strains.

Ribosomal multilocus sequence typing employing 53 full-length gene sequences encoding bacterial ribosome protein subunits (*rps*) was used to assess phylogenetic relationships between novel strain T173^T^ and 17 type strains of *Ensifer* species (Jolley et al., 2012). The Genome Comparator tool in the bacterial domain genome database of the BIGSdb software platform (Jolley and Maiden, 2010) was used to retrieve aligned concatenated sequences of *rps* genes from the genome sequences of novel strain T173^T^ and reference strains. The best-fit substitution model was selected using ModelTest-NG (Darriba et al., 2020).

A phylogenomic tree of strain T173^T^ and species type strains of the genus *Ensifer* was inferred with FastME 2.1.6.1 (Lefort et al., 2015) from Genome Blast Distance Phylogeny (GBDP) distances calculated from whole genome sequences using the suite of algorithms implemented in the TYGS (Type Strain Genome Server) web-based server (Meier-Kolthoff and Göker, 2019).

## Results and discussion

### Analyses of partial gene sequences

**Figure 1:**
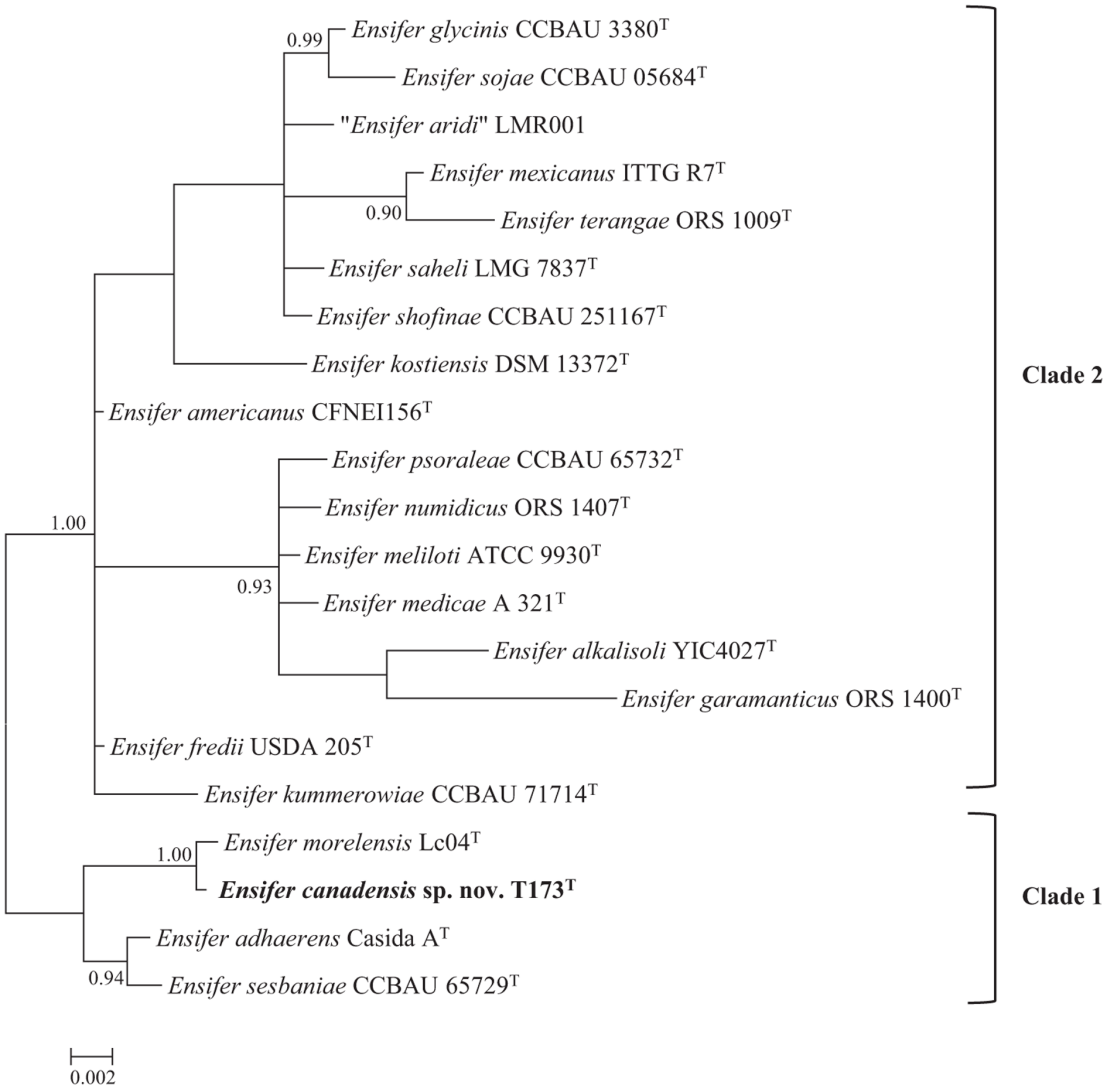
Bayesian phylogenetic tree of 16S rRNA gene sequences (1,401 bp) of *Ensifer canadensis* sp. nov. T173^T^ and reference taxa (HKY + G + I substitution model). Only posterior probabilities ≥90% are shown. Scale bar represents expected number of substitutions per site.

The 16S rRNA gene is universally present in all bacteria and sequences of this gene represent the most common house-keeping genetic marker that has been used in studies of bacterial taxonomy (Janda and Abbott, 2007). To reconstruct a 16S rRNA gene tree of type strains of all described *Ensifer* species (Supplementary Table S1) it was necessary to trim aligned sequence lengths to 1,401 bp. The Bayesian phylogenetic tree of 16S rRNA gene sequences (Figure 1) confirms placement of novel strain T173^T^ in the genus *Ensifer* and shows division of species type strains into two highly supported clades (labelled 1 and 2) represented by the type strains of *E. adhaerens* and *E. meliloti*, respectively. These two clades correspond to the “non-symbiotic” and “symbiotic” clades defined by Fagorzi et al. (2020) and Kuzmanović et al. (2022) on the basis of genomic and phenotypic analyses. Figure 1 further shows that novel strain T173^T^ is placed in the clade represented by *E. adhaerens* (clade 1), with the type strain of *E. morelensis* as closest relative.

As a cautionary note, it should be pointed out that the 16S rRNA gene is highly conserved and as such has limited usefulness as a taxonomic tool for delineating bacterial species (Richter and Rosselló-Móra, 2009; de Lajudie et al., 2019).

**Figure 2:**
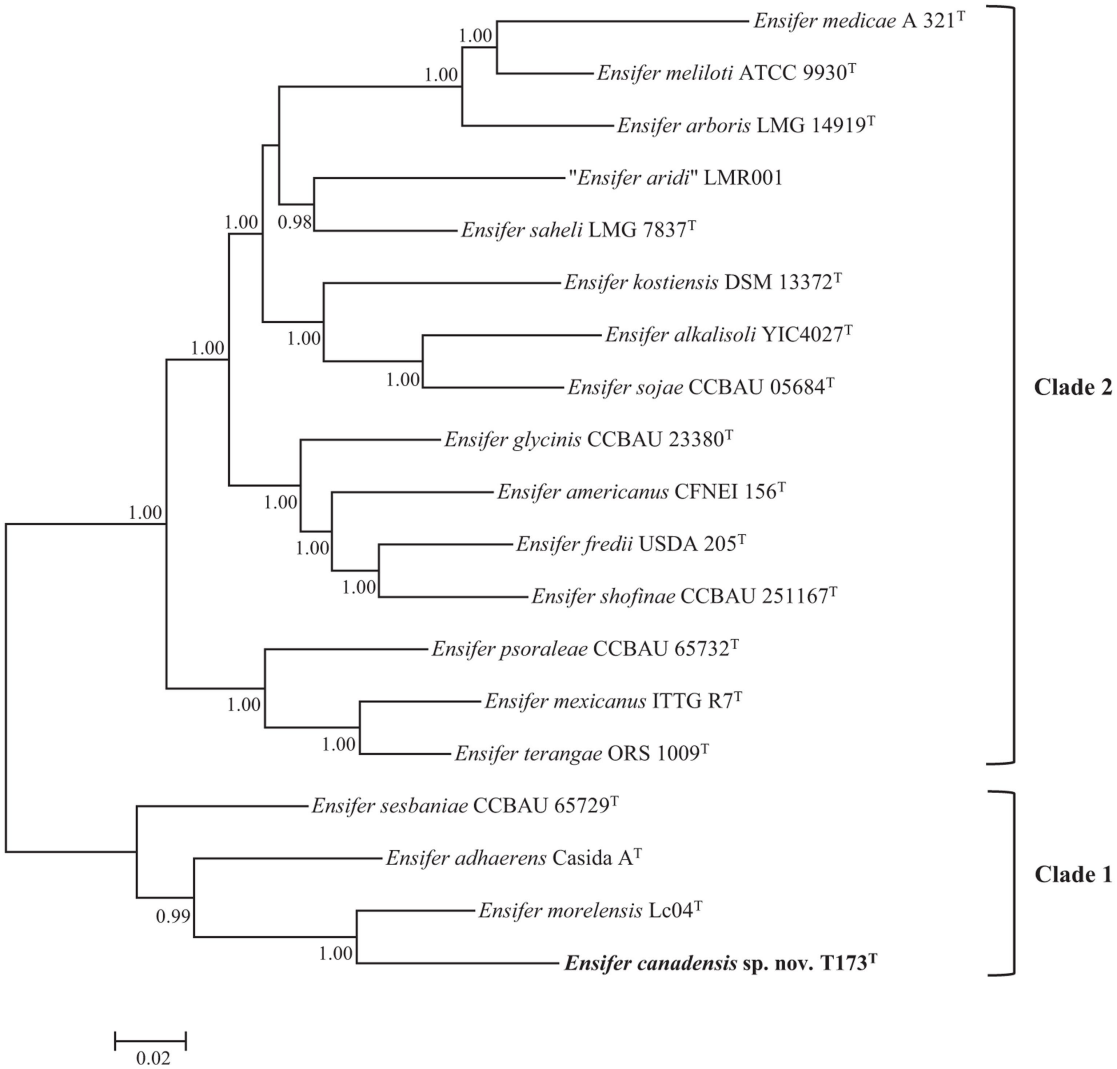
Bayesian phylogenetic tree (GTR + G + I substitution model) of *atpD-glnII-recA-gyrB-rpoB* concatenated partial housekeeping gene sequences (6,096 bp) of *Ensifer canadensis* sp. nov. T173^T^ and reference taxa of the genus *Ensifer*. Only posterior probabilities ≥90% are shown. Bar, expected substitutions per site.

Phylogenetic analysis of multiple protein encoding (house-keeping) partial gene sequences (Multiple Locus Sequence Analysis, MLSA) has often been used to facilitate bacterial species delineation (e.g., Martens et al., 2008; de Lajudie et al., 2019). The topology of the Bayesian tree of five concatenated protein encoding (*atpD*, *glnII*, *gyrB*, *recA*, and *rpoB*) gene sequences (Figure 2), confirms the division of 18 species type strains of the genus *Ensifer* into two highly supported clades. Figure 2 also confirms placement of strain T173^T^ in a highly supported lineage that is distinct from type strains of *Ensifer* species with *E. morelensis* as closest relative.

As one or more housekeeping gene sequences of several type strains of *Ensifer* species are not available in public databases, we used sequences of the *glnII* gene (the only protein encoding house-keeping gene for which sequences are available for type strains of 21 *Ensifer* species) in a supplementary phylogenetic analysis to verify the uniqueness of strain T173^T^. To include all 21 type strains in the phylogenetic tree it was necessary to trim the aligned lengths of *glnII* gene sequences to 615 bp. Consistent with the topology of trees of 16S rRNA (Figure 1) and five protein encoding (Figure 2) gene sequences, the tree of partial *glnII* sequences (Supplementary Figure S1) shows placement of strain T173^T^ in a highly supported lineage distinct from all 21 described species of the genus *Ensifer*.

### Genome analyses

A complete circularized genome sequence of strain T173^T^ was generated in this study using PacBio Sequel technology. Estimated genome coverage was 419-fold with 96,490 polymerase reads and an average read length of 37,345 bp. Data for genome characteristics of strain T173^T^ and reference strains of *Ensifer* for which complete genome sequences are available are shown in Table 1. The size of the complete genome sequence of strain T173^T^ is 8,094,229 bp with an average DNA G + C content of 61.0 mol%. Six circularized replicons corresponding to a chromosome and five plasmids (pT173a through pT173e) were detected in the genome sequence of T173^T^. All plasmids harboured *repABC* genes encoding proteins involved in plasmid replication and segregation (Pinto et al., 2012); plasmid pT173c harboured two complete *repABC* gene copies. The six replicons of strain T173^T^ have the following sizes (bp): 195,834 (pT173a); 204,278 (pT173b); 782,207 (pT173c); 946,878 (pT173d); 1,913,930 (pT173e), and 4,051,102 bp (chromosome). These data for plasmid number and size are consistent with the results of plasmid profile analysis (horizontal agarose gel electrophoresis) of strain T173^T^ in our earlier study (Bromfield et al., 2010): four plasmid bands were clearly resolved with the fourth band (exhibiting greatest mobility) likely representing a doublet consisting of co-migrating plasmids pT173a and pT173b with closely similar sizes.

**Table 1:** Characteristics of complete genome sequences of *Ensifer canadensis* sp. nov. T173^T^ and reference strains.

Characteristic	Strain						
	*E. canadensis* sp. nov. T173^T^	*E. adhaerens* Casida A^T^	*E. alkalisoli* YIC4027^T^	*E. medicae* WSM1115	*E. meliloti* 1021	*E. mexicanus* ITTG R7^T^	*E. sojae* CCBAU 05684^T^
Genome assembly quality (no. replicons)	Complete (6)	Complete (3)	Complete (3)	Complete (4)	Complete (3)	Complete (4)	Complete (3)
Genome size (bp)	8,094,229	7,267,502	6,128,433	7,063,185	6,691,694	7,141,863	6,094,027
Chromosome size (bp)	4,051,102	4,071,185	3,690,234	4,106,266	3,654,135	4,316,340	3,672,259
Plasmid size (bp)*	195,834; 204,278; 782,207; 946,878; 1,913,930	1,459,374; 1,736,943	456,454; 1,981,775	276,847; 1,128,391; 1,551,681	1,354,226; 1,683,333	436,172; 455,676; 1,933,675	410,255; 2,011,513
Genes (total)	7,705	6,937	5,797	6,832	6,314	6,641	5,764
CDSs (total)	7,625	6,854	5,536	6,763	6,267	6,574	5,699
Plasmids (*repABC* copies)	5 (6)	2 (2)	2 (2)	3 (3)	2 (2)	3 (3)	2 (2)
G + C content % (Entire genome sequence)	61.0	62.3	62.2	61.2	62.2	61.4	62.0
G + C content % (Chromosome)	61.8	62.8	62.6	61.5	62.7	61.8	62.4
G + C content % (Megaplasmids*)	60.1; 61.2^†^	60.3; 62.8	62.3	59.9; 61.6	60.4; 62.4^†^	61.6	61.9
G + C content % (Plasmids*)	58.0; 58.4; 58.6		59.3	60.6		58.6; 60.2	59.0
Replicon (rRNA operon copies)	Chromosome (3); pT173d (1); pT173e (1)	Chromosome (3); pCasidaAA (1); pCasidaAB (1)	Chromosome (3)	Chromosome (3)	Chromosome (3)	Chromosome (3)	Chromosome (3)
Symbiosis plasmid (pSym)	pT173b (204,278 bp)	none	Accession no: CP034911 (456,424 bp)	pWSM1115_2 (1,128,391 bp)	pSymA (1,354,226 bp)	pEmeITTGR7b (455,676 bp)	pSJ05684a (410,255 bp)
Antibiotic Resistance Genes	51	42	39	40	45	43	37
tRNAs	61	64	56	56	55	54	52

*Data are presented in order of increasing replicon size.

^†^Chromid.

In many bacterial genomes the ribosomal RNA (*rrn*) operon (encoding 16S, 23S, and 5S rRNAs) exists in multiple copies that are usually restricted to the chromosome (Acinas et al., 2004; Pei et al., 2010; Espejo and Plaza, 2018). Consistent with these reports multiple *rrn* copies were found on the chromosome of strain T173^T^ and all *Ensifer* reference strains listed in Table 1. Of the five *rrn* copies detected in strain T173^T^, three were on the chromosome and single copies were on the large plasmids (megaplasmids), pT173d and pT173e. The closely related strain *E. adhaerens Casida A*^T^ also possessed single *rrn* copies on megaplasmids (pCasidaAA and pCasidaAB) as well as three copies on the chromosome. The presence of *rrn* copies on both bacterial chromosomes and plasmids is not without precedence and has been found in *Vibrio parahaemolyticus* (Yamaichi et al., 1999), *Bacillus megaterium* (Kunnimalaiyaan et al., 2001) and soil isolates of *Paracoccus* species (Battermann et al., 2003). Moreover, in one unusual case a strain of *Aureimonas* sp. was reported to have its sole *rrn* operon on a small (9.4 kb) plasmid instead of the chromosome (Anda et al., 2015).

The occurrence of genetic heterogeneity among multiple *rrn* operons within genomes is well documented in bacteria (Yap et al., 1999; Tourova et al., 2001; Acinas et al., 2004).

Of the seven *Ensifer* strains in Table 1, only strain T173^T^ and *E. medicae* strain WSM115 were found to exhibit intra-genomic variation in *rrn* copies. For strain T173^T^ the two *rrn* copies on megaplasmids pT173d and pT173e differed (at the 23S rRNA locus) from the three copies on the chromosome whereas of the three *rrn* copies on the chromosome of *E. medicae* WSM115, one copy differed at the 16S rRNA locus and a second copy differed at the 23S rRNA locus. In all cases there were < 1% nucleotide differences between operons which is consistent with the levels of intra-genomic divergence reported for most bacteria (Acinas et al., 2004).

The finding that the very large megaplasmid, pT173e, of strain T173^T^ exhibited a G + C content value (61.2%) that was similar to the chromosome (61.8%), together with the presence of *repABC* genes and housekeeping genes (*rrn* operon) (Table 1) suggests that this plasmid may represent a “chromid” (secondary chromosome) as defined by Harrison et al. (2010). Consistent with other studies (Nishida, 2012), smaller plasmids in the genome of strain T173^T^ (i.e., plasmids other than the “chromid”) as well as those in the genomes of *Ensifer* reference strains (Table 1), consistently show G + C contents (58.0–60.25%) that are lower than that of the respective chromosomes (61.5–62.8%), suggesting that these plasmids may have been acquired from external sources by horizontal genetic exchange (Garcia-Vallvé et al., 2000).

**Figure 3:**
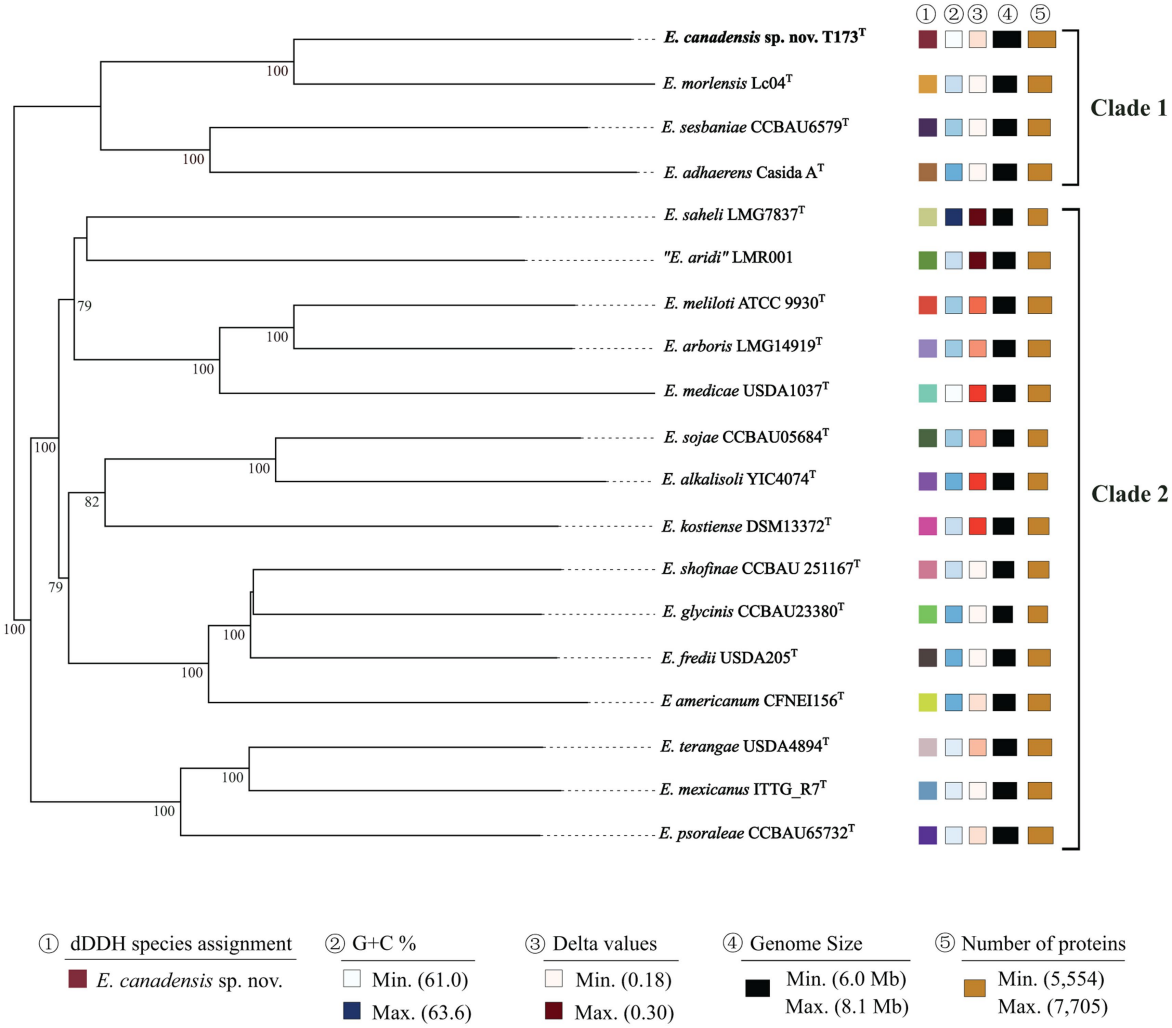
Phylogenomic tree based on Type Strain Genome Server (TYGS) implementation showing *E. canadensis* sp. nov. T173^T^ and reference taxa (species type strains) of the genus *Ensifer*. The tree was inferred with FastME 2.1.6.1 (Lefort et al., 2015) from GBDP distances calculated from genome sequences. The branch lengths are scaled in terms of GBDP distance formula d5. The numbers above branches represent GBDP pseudo-bootstrap support values ≥70% from 100 replications, with an average branch support of 95.7%. Leaf labels are annotated by affiliation to species (1), genomic G + C content (2), delta values (3), overall genome sequence length (4), and number of proteins (5). Delta statistics permit assessment of accuracy in terms of tree-likeness; the lower the delta value, the greater the accuracy.

To further verify the taxonomic status of strain T173^T^ relative to type strains of *Ensifer* species, we carried out phylogenetic analyses based on: (1) MLSA of 53 concatenated full-length house-keeping gene sequences encoding bacterial ribosome protein subunits (*rps*) (Jolley et al., 2012), and, (2) TYGS analysis of whole genome sequences (Meier-Kolthoff and Göker, 2019). The topology of the phylogenetic tree of *rps* gene sequences (Supplementary Figure S2) as well as the TYGS tree of whole genome sequences (Figure 3) corroborate our findings that strain T173^T^ is consistently placed in a highly supported lineage distinct from type strains of described species of *Ensifer* with *E. morelensis* as closest relative.

Data for dDDH and ANI values for pair-wise comparisons of genome sequences of novel strain T173^T^ with the three closest relatives (i.e., all species in the *E. adhaerens* clade) are presented in Supplementary Table S2. The highest dDDH and ANI values (35.7 and 88.6%, respectively) obtained in these comparisons are well below the respective threshold values of 70% and 95–96% for bacterial species circumscription. Based on these data, strain T173^T^ is unambiguously classified as a novel *Ensifer* species with *E. morelensis* as closest relative.

**Figure 4:**
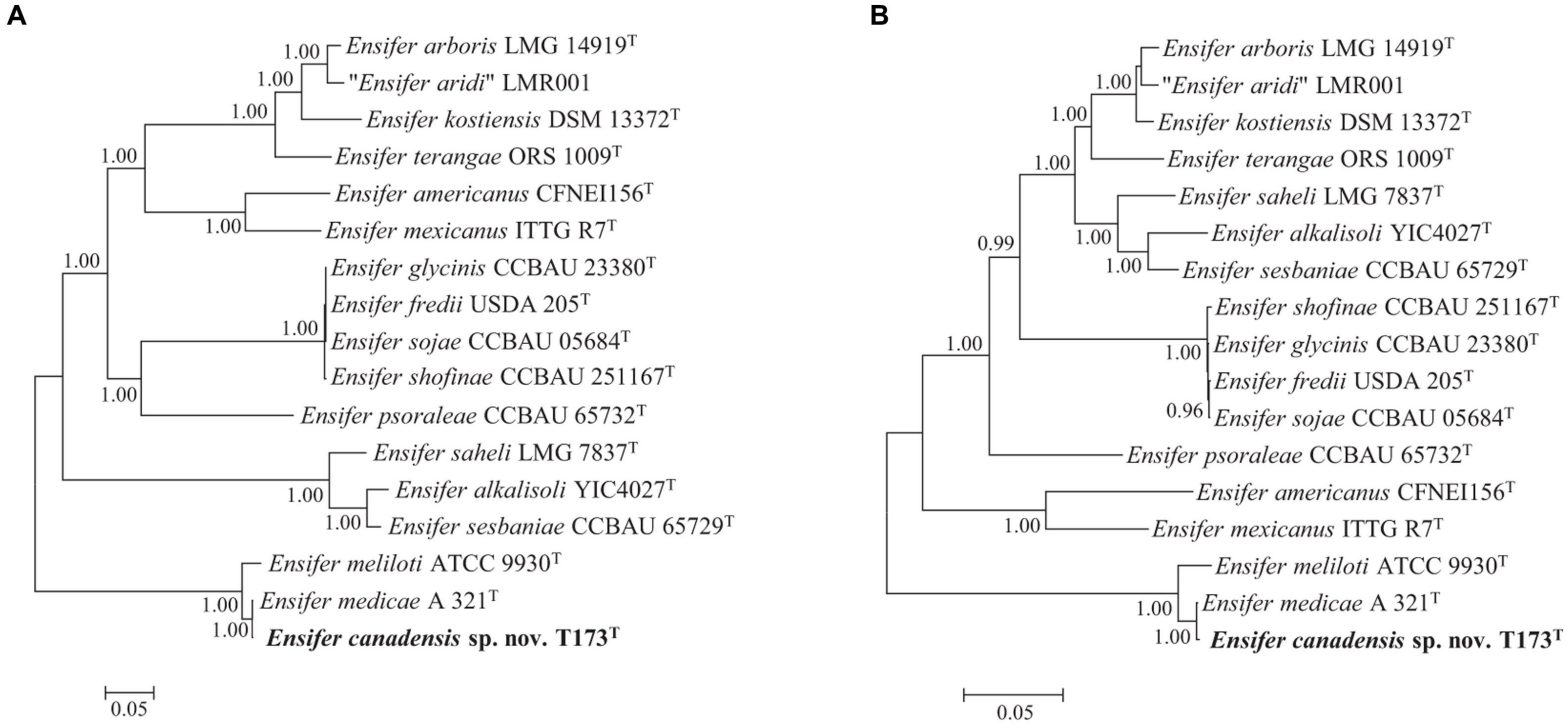
Bayesian phylogenetic trees (HKY + G + I substitution model) of **(A)** *nodABC* concatenated gene sequences (2,352 bp), and **(B)** *nifHDK* concatenated gene sequences (3,828 bp) of *Ensifer canadensis* sp. nov. T173T and reference taxa of the genus *Ensifer*. Only posterior probabilities ≥90% are shown. Bar, expected substitutions per site.

Consistent with our earlier study (Bromfield et al., 2010), key nodulation (*nod*) and nitrogen fixation (*nif*) genes were detected on plasmid pT173b, representing the symbiosis plasmid (or pSym). To investigate the evolutionary history of symbiosis genes on pT173b we reconstructed Bayesian phylogenetic trees of concatenated *nodABC* and *nifHDK* full length gene sequences of strain T173^T^ and 16 species type strains of the genus *Ensifer*. The topologies of the *nodABC* and *nifHDK* gene trees (Figures 4A,B) are closely similar with strain T173^T^ and the type strain of *E. medicae* (possessing almost identical *nod* and *nif* gene sequences) placed together in the same lineage. These results suggest that strain T173^T^ placed in the *E. adhearens* clade in the house keeping gene and genome trees (e.g., Figures 2, 3), has acquired its symbiosis genes by horizontal transfer from *E. medicae* placed in the phylogenetically distant *E. melitoti* clade in Figures 2, 3.

**Figure 5:**
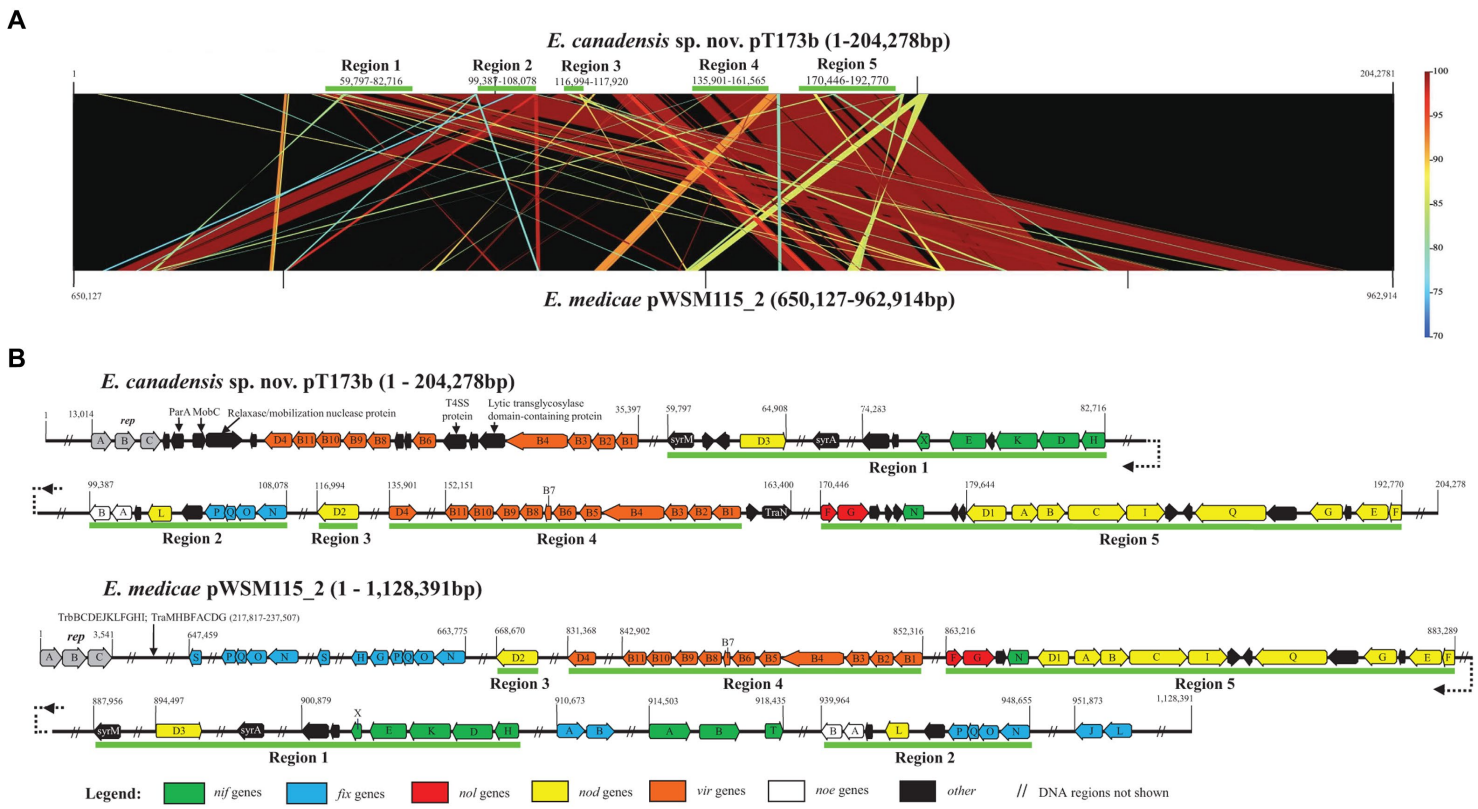
Comparison of the sequence of symbiosis plasmid pT173b with the symbiosis gene region of the pSymA megaplasmid (=pWSM1115_2) of *Ensifer medicae* WSM1115 showing **(A)** five regions of the pT173b sequence that exhibit high sequence similarity (>98%) with genes on pWSM1115_2 and **(B)** detail of regions 1 through 5 harbouring nodulation (*nod*, *noe*, and *nol*), nitrogen-fixation (*nif* and *fix*) and type IV secretion system (T4SS) genes.

To further investigate the apparent horizontal acquisition of symbiosis and related genes by strain T173^T^ we compared the complete sequence of symbiosis plasmid pT173b with the symbiosis gene region of the pSymA megaplasmid (=pWSM1115_2) of *E. medicae* WSM1115, for which a complete genome sequence is available; pWSM1115_2 was selected based on the best BLAST hit using the complete sequence of pT173b as query. Figure 5A shows that five regions of the pT173b sequence contain symbiosis and related genes that exhibit high sequence similarity (>98%) to genes on pWSM1115_2. Regions 1, 2, 3, and 5 consist of nodulation (*nod*, *noe*, and *nol*) and nitrogen-fixation (*nif* and *fix*) genes that were apparently acquired from *E. medicae* (Figure 5B). However, key genes required for nitrogen-fixation such as *nifA*, *nifB*, *fixLJ*, *fixK*, and *fixGHIS* (Lindström and Mousavi, 2020) present in the sequence of pWSM1115_2 (*E. medicae*) were not found in the sequence of pT173b. Therefore, it was not unexpected to find that that strain T173^T^ did not fix nitrogen in association with any of the host legumes tested in this and in the previous study (Bromfield et al., 2010). Region 4 of the pT173b sequence (Figure 5B), apparently acquired from *E. medicae*, contains genes encoding a conjugative type IV secretion system (T4SS) (Alvarez-Martinez and Christie, 2009; Rudder et al., 2014) that is incomplete and lacks a relaxase gene (Trokter and Waksman, 2018) necessary for conjugation. A second conjugative T4SS as well as plasmid replication and segregation (*repABC*) genes were detected on pT173b that are not shared with pWSM115_2. BLAST searches of the T4SS gene segment, *virB1* through *virB11* (pT173b co-ordinates: 23916–35,397 bp) and the *repABC* gene segment (pT173b co-ordinates 13,014–16,648 bp) against NCBI genome sequence databases (Supplementary Table S3) suggest that these genes are most closely related to replication and conjugation systems in *Neorhizobium galegae*.

Collectively, these findings suggest that symbiosis plasmid pT173b is a recombinant plasmid with plasmid replication and conjugative T4SS gene regions that have their closest relatives in species like *N. galegae* and large segments of DNA containing symbiosis and T4SS genes that apparently originated from a symbiosis megaplasmid of *E. medicae*. It is noteworthy that host plants of *E. medicae* (*Medicago* and *Melilotus* species) and *N. galegae* (*Galega officinalis* and *Galega orientalis*) are native to the same region of Eurasia (Mediterranean basin/Caucasus) (Quiros and Bauchan, 1988; Rome et al., 1996; Steele et al., 2010; Ramírez-Bahena et al., 2015; Darbyshire et al., 2021) suggesting that the hybrid symbiosis plasmid, pT173b, may have had its origins in Eurasia.

Plasmid conjugation systems represent important mechanisms for the horizontal transmission of genetic information such as symbiotic lifestyle and antibiotic resistance traits that facilitate the rapid evolution and adaptation of bacteria to new or changing environments (Chen et al., 2022). We detected genes involved in conjugation on four of the five plasmids in strain T173^T^ (plasmids pT173a, b, c, and e). The plasmid conjugation systems in species of *Rhizobium*, *Agrobacterium* and *Ensifer,* as well as other members of the *Rhizobiaceae*, are classified into at least four different types (Ding and Hynes, 2009; Ding et al., 2013). Sequence comparisons of the conjugation systems and analysis of gene synteny in plasmids of strain T173^T^ with well characterized members of each type of conjugation system show that pT173a carries a typical type I system, such as is found in *Agrobacterium* Ti plasmids, and is most likely regulated by quorum sensing as indicated by the presence of an autoinducer synthase gene (*traI*) and a *traR* orthologue (Gordon and Christie, 2014). The large plasmid or “chromid,” pT173e, was found to encode a complete type II system, similar to those found on plasmid pCFN42d of *Rhizobium etli* strain CFN42 and the pSymA plasmid of *Ensifer meliloti* strain 1021; it is common for this type of system, or a reduced version of it, to be present on megaplasmids or chromids of *Ensifer* species (Pérez-Mendoza et al., 2005; Blanca-Ordóñez et al., 2010). Two conjugation systems were detected on the symbiosis plasmid, pT173b, but one of these lacks a relaxase gene (as described above) and may not be functional. The second is similar to the system encoded on pT173c, and most closely resembles type IVb systems as described by Ding et al. (2013). We compared the relaxase (MobZ or TraA), coupling protein (usually designated TraG or VirD4) and best conserved protein from the T4SS (designated TrbE or VirB4, depending on the system) between these conjugation gene clusters on pT173b and pT173c and found that there is 62% identity between the relaxases (J3R84_28450 on pT173b and J3R84_31165 on pT173c), 77% identity between the coupling proteins (J3R84_28460 and J3R84_30945, respectively), and 91% identity between the VirB4/TrbE proteins (J3R84_28515 and J3R84_30995, respectively). These conjugation gene clusters on pT173b and pT173c are organized like those on plasmid pAtS4a of *Agrobacterium vitis* strain S4 and plasmid pSmed03 of *Ensifer medicae* strain WSM419 (Ding et al., 2013), which were among the earliest recognized members of the type IVb system (Giusti et al., 2012).

Based on our genome analyses, it seems likely that four of the plasmids of T173^T^ are mobile. As such, strain T173^T^ represents a valuable resource for studies on plasmid self transmissibility. Indeed, mobile plasmids are highly prevalent in the *Rhizobiaceae*, and there appears to be strong selection for mobility of symbiosis genes between strains (Wardell et al., 2022). This selection for transmission of plasmids harbouring symbiosis genes might explain the common occurrence of mosaic plasmids (Wardell et al., 2022), arising from recombination between segments of multiple plasmids, as seems to have been the case for the symbiosis plasmid, pT173b of strain T173^T^.

### Phenotypic characterization

Colonies of strain T173^T^ are raised, mucilaginous, circular and off-white coloured with diameters ~2–3 mm after 3 days growth at 28°C on yeast extract-mannitol (YEM) agar medium. Bacterial cells are Gram-stain-negative rods and based on electron microscopy exhibit multiple (peritrichous) flagella (Supplementary Figure S3).

Data for growth characteristics of strain T173^T^ and seven reference strains of the genus *Ensifer* are shown in Supplementary Table S4. Strain T173^T^ produces an acidic reaction on YEM agar after 21 days growth at 28°C typical of other members of the genus *Ensifer*. Strain T173^T^, like close relatives, *E. morelensis* Lc04^T^ and *E. adhaerens* Casida A^T^, shows growth in the presence of 2% NaCl, at pH 5 and pH10, and, at temperatures of 10°C and 37°C on YEM agar after 2 days incubation. Strain T173^T^ shows good growth in LB broth medium after 48 h at 30°C (Supplementary Table S4; Supplementary Figure S4) which is considered typical of members of the *E. adhaerens* clade (Fagorzi et al., 2020). Unlike *E. adhaerens* Cassida A^T^, novel strain T173^T^ and closest relative, *E. morelensis* Lc04^T^, did not exhibit tracking activity (i.e., predation) (Martin, 2002) of *Micrococcus luteus* JCM 1464^T^ grown on 0.1x heart infusion agar medium supplemented with 0.1% glucose at 28°C after 7 days.

The results of 70 carbon source utilization and 18 chemical sensitivity tests using phenotype microarrays (Biolog) are given in Supplementary Table S5. On the basis of multiple tests, strain T173^T^ could be readily differentiated from all seven type strains of *Ensifer* species that we tested. In particular, T173^T^ could be distinguished from its closest relative, *E. morelensis* Lc04^T^, based on differential utilization of 20 carbon sources and sensitivity to three chemical compounds. Moreover, in our previous study (Bromfield et al., 2010), strain T173^T^ was shown to be unusual in that it was highly resistant to multiple antibiotics including carbenicillin (>1,000 μg ml^−1^), kanamycin (>100 μg ml^−1^) and neomycin (~100 μg ml^−1^), similar to its close relative, *E. morelensis* Lc04^T^ (Wang et al., 2002). In this connection, multiple antibiotic resistance genes (Yoneyama and Katsumata, 2006) were detected in the genome of strain T173^T^ (Table 1) including genes encoding enzymes that inactivate beta-lactam antibiotics (e.g., carbenicillin) and amino-glycoside antibiotics such as kanamycin and neomycin (Supplementary Table S6).

Data for the fatty acid profiles of T173^T^ and reference strains are shown in Supplementary Table S7. Consistent with the results of other studies of the genus *Ensifer* (Tighe et al., 2000), fatty acids 16:0, 18:0, 18:0 3OH, 19:0 cyclo ω8c, 12:0 aldehyde/? (summed feature 2) and 18:1 ω6c/18:1 ω7c (summed feature 8) were common to strain T173^T^ and reference strains of seven *Ensifer* species. Fatty acid 18:1 ω7c 11-methyl was predominant (>15%) only in strain T173^T^ and close relatives, *E. morelensis* Lc04^T^ and *E. adhaerens* Casida A^T^. It is noteworthy that the overall profile (24 fatty acids including minor fatty acids) distinguished strain T173^T^ from all seven reference strains of *Ensifer* species.

Plant tests in this work and in the previous study (Bromfield et al., 2010) showed that strain T173^T^ elicited numerous small white nodules on roots of *Medicago sativa* (alfalfa), *Melilotus albus* (white sweet clover), *Medicago polymorpha* (burr medic) and *Macroptilium atropurpureum* (siratro), but did not fix nitrogen.

### Description of *Ensifer canadensis* sp. nov.

*Ensifer canadensis* sp. nov. *ca.*na.den’sis. N.L. masc./fem. Adj. canadensis, of or belonging to Canada, from where the organism was isolated.

Cells are Gram-stain-negative, aerobic, non-spore-forming rods with multiple flagella. The type strain produces colonies that are raised, mucilaginous, circular and off-white coloured with diameters ~2–3 mm after 3 days growth at 28°C on YEM agar medium.

Grows in the presence of 2% NaCl and at pH 5 and pH10 (optimum ~pH 6.0–7.0) after 2 days at 28°C on YEM agar medium. The type strain grows at temperatures of 10°C and 37°C (optimal at ~28°C) after 2 days on YEM agar. Grows in LB broth medium after 2 days at 30°C. Produces an acidic reaction on YEM agar after 21 days growth at 28°C. Does not show predatory activity against *Micrococcus luteus* after 7 days incubation at 28°C on 0.1x heart infusion agar medium supplemented with 0.1% glucose. Predominant fatty acids are 16:0, 18:0, 18:1 ω7c 11-methyl, 12:0 aldehyde/? (summed feature 2) and 18:1 ω6c/18:1 ω7c (summed feature 8).

The type strain utilizes 35 carbon sources including sucrose, D-raffinose, α-D-lactose, N-acetyl-β-D-mannosamine, D-mannose, L-fucose, glycerol, D-fructose- 6-PO4, D-aspartic acid, L-glutamic acid, pectin, D-gluconic acid, D-glucuronic acid, propionic acid and formic acid. Does not utilize 35 carbon sources including dextrin, stachyose, β-methyl-D-glucoside, D-salicin, N-acetyl-D-galactosamine, D-galactose, inosine, glycyl-L-proline, L-arginine, L-aspartic acid, L-histidine, L-serine, D-lactic acid methyl ester, L-lactic acid, and citric acid. Resistant to troleandomycin, rifamycin SV, lincomycin, tetrazolium violet, tetrazolium blue and aztreonam. Susceptible to 12 chemical compounds including 1% sodium lactate, guanidine HCl, niaproof 4, vancomycin, nalidixic acid and potassium tellurite. The type strain is highly resistant to carbenicillin (>1,000 μg ml^−1^), kanamycin (>100 μg ml^−1^) and neomycin (~100 μg ml^−1^).

The type strain elicits numerous small white nodules (ineffective for nitrogen fixation) on roots of *Medicago sativa*, *Medicago lupulina*, *Medicago polymorpha*, *Melilotus albus*, and *Macroptilium atropurpureum*.

The type strain, T173^T^ (= LMG 32374^T^ = HAMBI 3766^T^) was isolated from a root-nodule of a *Melilotus albus* plant grown at a field site in Ottawa, Ontario, Canada. The whole genome shotgun project for *Ensifer canadensis* strain T173^T^ was deposited at DDBJ/ENA/GenBank under the accession numbers CP083370–CP083375. Raw PacBio data was deposited in the NCBI Sequence Read Archive under the BioProject accession number PRJNA713338. The genome of the type strain contains a chromosome and five plasmids one of which is a symbiosis plasmid harbouring nodulation and nitrogen fixation genes. The DNA G + C content of the type strain is 61.0 mol% and the genome size is 8,094,229 bp.

## Data availability statement

The datasets presented in this study can be found in online repositories. The names of the repository/repositories and accession number(s) can be found in the article/Supplementary material.

## Author contributions

EB co-ordinated the project, received the funding, and wrote the draft manuscript. SC and EB carried out the experiments. SC, EB, and MH analyzed the data. All authors contributed to the article and approved the submitted version.

## Funding

This research was supported by grants J-002272 and J-002295 from Agriculture and Agri-Food Canada.

## Conflict of interest

The authors declare that the research was conducted in the absence of any commercial or financial relationships that could be construed as a potential conflict of interest.

## Publisher’s note

All claims expressed in this article are solely those of the authors and do not necessarily represent those of their affiliated organizations, or those of the publisher, the editors and the reviewers. Any product that may be evaluated in this article, or claim that may be made by its manufacturer, is not guaranteed or endorsed by the publisher.

## Footnotes

^1^ https://www.geneious.com
