# Supplementary material for: Ensifer canadensis sp. nov. strain T173T isolated from Melilotus albus (sweet clover) in Canada possesses recombinant plasmid pT173b harbouring symbiosis and type IV secretion system genes apparently acquired from Ensifer medicae
Source: Front Microbiol. 2023 Jun 14;14:1195755. doi: 10.3389/fmicb.2023.1195755 (PMC10306167; doi:10.3389/fmicb.2023.1195755)
Supplement: Supplementary file 1 [file Data_Sheet_1.PDF]

Supplementary Material

Ensifer canadensis sp. nov strain T173^T isolated from *Melilotus albus* (sweet clover) in Canada possesses recombinant plasmid pT173b harbouring symbiosis and Type IV secretion system genes apparently acquired from *Ensifer medicae*

Eden S. P. Bromfield*, Sylvie Cloutier and Michael F. Hynes

***Correspondence:** Eden S. P. Bromfield, eden.bromfield@agr.gc.ca

Supplementary Table 1. GenBank sequence accession numbers of *Ensifer canadensis* sp. nov. T173^T and reference taxa used in this study.

Strain	*atpD*	*glnII*	*gyrB*	*recA*	*rpoB*	*16S rRNA*	*16S-23S-5S rRNA*	*nifHDK*	*nodABC*
***Ensifer canadensis* sp. nov. T173^T**	CP083370	CP083370	CP083370	CP083370	CP083370	CP083370	CP083370<br>CP083371<br>CP083372	CP083372	CP083372
Ensifer adhaerens Casida A^T	CP015880	CP015881	CP015880	CP015880	CP015880	CP015880	CP015880<br>CP015881<br>CP015882	na	na
Ensifer alkalisoli YIC4027^T	CP034909	CP034910	CP034909	CP034909	CP034909	CP034909	CP034909	CP034911	CP034911
Ensifer americanus CFNEI156^T	LNQC01	LNQC01	LNQC01	LNQC01	LNQC01	LNQC01	LNQC01	LNQC01	LNQC01
Ensifer arboris LMG 14919^T	ATYB01	HM997093	ATYB01	ATYB01	ATYB01	‡ATYB01	ATYB01	ATYB01	ATYB01
"*Ensifer aridi*" LMR001	LUAV01	LUAV01	LUAV01	LUAV01	LUAV01	LUAV01	LUAV01	KP993264	LUAV01
"*Ensifer collicola*" Mol12	-	-	-	-	-	*KT724704	-	-	-
Ensifer fredii USDA 205^T	WISZ01	WISZ01	WISZ01	WISZ01	WISZ01	WISZ01	WISZ01	WISZ01	WISZ01
Ensifer garamanticus ORS 1400^T	-	HM997091	-	-	-	AY500255	-	-	-
Ensifer glycinis CCBAU 23380^T	LPUX01	LPUX01	LPUX01	LPUX01	LPUX01	LPUX01	LPUX01	LPUX01	LPUX01
Ensifer kostiensis DSM 13372^T	JAGILA01	JAGILA01	JAGILA01	JAGILA01	JAGILA01	JAGILA01	JAGILA01	JAGILA01	JAGILA01
Ensifer kummerowiae CCBAU 71714^T	-	GU994062	-	-	-	AY034028	-	-	-
Ensifer medicae A 321^T	VITA01	VITA01	VITA01	VITA01	VITA01	VITA01	VITA01	VITA01	VITA01
Ensifer medicae WSM1115	na	na	na	na	na	na	CP088109	na	na
Ensifer meliloti ATCC 9930^T	BJNJ01	BJNJ01	BJNJ01	BJNJ01	BJNJ01	BJNJ01	BJNJ01	BJNJ01	BJNJ01
Ensifer meliloti 1021	na	na	na	na	na	na	AL591688	na	na
Ensifer mexicanus ITTG R7^T	CP041238	CP041241	CP041238	CP041238	CP041238	CP041238	CP041238	CP041240	CP041240
Ensifer morelensis Lc04^T	JABEKT01	HM997095	JABEKT01	JABEKT01	JABEKT01	JABEKT01	JABEKT01	-	-
Ensifer numidicus ORS 1407^T	-	HM997092	-	-	-	AY500254	-	-	-
Ensifer psoraleae CCBAU 65732^T	JABEKV01	JABEKV01	JABEKV01	JABEKV01	JABEKV01	JABEKV01	JABEKV01	JABEKV01	JABEKV01
Ensifer saheli LMG 7837^T	LNQB01	LNQB01	LNQB01	LNQB01	LNQB01	LNQB01	LNQB01	LNQB01	LNQB01
Ensifer sesbaniae CCBAU 65729^T	JABEKU01	JABEKU01	JABEKU01	JABEKU01	JABEKU01	JABEKU01	JABEKU01	JABEKU01	JABEKU01
Ensifer shofinae CCBAU 251167^T†	MBFK01	MBFK01	MBFK01	MBFK01	MG646990	MBFK01	MBFK01	MBFK01	MBFK01
Ensifer sojae CCBAU 05684^T	AJQT01	AJQT01	AJQT01	AJQT01	AJQT01	AJQT01	AJQT01	AJQT01	AJQT01
Ensifer terangae ORS 1009^T	WITC01	WITC01	WITC01	WITC01	WITC01	WITC01	WITC01	WITC01	WITC01

na, Not applicable; - Sequence not available or too short.
* The 16S rRNA gene sequence (KT724704) of "*E. collicola"* Mol 12 was an outlier and excluded from phylogenetic analyses.
† *E. shofinae* CCBAU 251167^T genome sequence MBFK01: NCBI notes "anomalous assembly"; *rpoB* (housekeeping gene) not found in genome sequence.
‡Three copies of the 16S rRNA gene present in the genome of *Ensifer arboris* LMG 14919^T (Accession no. ATYB01) were found to be anomalous and excluded from the 16S rRNA phylogenetic analysis.

Supplementary Table 2. Average Nucleotide Identity (ANI) and digital DNA–DNA hybridization (dDDH) values for pair-wise comparisons of genome sequences of *Ensifer canadensis* sp. nov. T173^{T} (Accession no. CP083370-CP083375) with closest relatives.

**Reference Strain (Sequence Accession no.)**	**Fast ANI***	**dDDH % [C.I.]†**
	**T173^{T}**	**T173^{T}**
Ensifer morelensis Lc04^{T} (JABEKT01)	88.6	35.7 [33.3-38.2]
Ensifer adhaerens Casida A^{T} (CP015880 - CP015882)	83.8	25.5 [23.2-28.0]
Ensifer sesbaniae CCBAU 65729^{T} (JABEKU01)	83.7	25.8 [23.5-28.3]

*Values represent averages of reciprocal comparisons.

†dDDH values and confidence intervals [C.I.] based on Genome BLAST Distance Phylogeny (GBDP) formula 4 implemented in the Type Strain Genome Server (TYGS); formula 4 is independent of genome length and is robust against use of incomplete draft genomes.

Supplementary Table 3. BLAST results for T4SS (*virB1* through *virB11*) and *repABC* genes of pT173b (CP083372) as query versus *Rhizobiales* whole-genome shotgun contigs and standard nr/nt databases in NCBI.

Query genes (co-ordinates on pT173b sequence)	Top BLAST hits				
	NCBI Database	ID (%)	Coverage (%)	Strain (Accession no.)	Comments
virB1 - virB11 (23916 - 35397 bp)	Whole-Genome Shotgun Contigs	92.48	100	*Neorhizobium galegae* bv. orientalis strain HAMBI 2605 (CCRM01)	NCBI notes: anomalous assembly
		91.17	100	*Neorhizobium galegae* strain NG_87_Ori (VZUL01)	Classification as *N. galegae* confirmed by phylogenomic analysis implemented in the Type Strain Genome Server (TYGS)
		90.80	99	*Rhizobiales* bacterium isolate AFS086343 (UCKS01)	
		90.80	100	*Rhizobium* sp. strain Root483D2 (LMFB01)	
		90.80	100	*Rhizobium* sp. strain Root268 (LMJD01)	
	Standard nr/nt	79.44	99	*Shinella* sp. PSBB067 plasmid unnamed (CP069305)	
		79.35	99	*Rhizobium daejeonense* strain KACC 13094 unnamed plasmid (CP048428)	
		79.14	99	*Shinella* sp. HZN7 plasmid pShin-06 (CP015742)	
		78.79	99	*Shinella zoogloeoides* strain ATCC 19623 unnamed plasmid (CP086613)	
repABC (13014 -16648 bp)	Whole-Genome Shotgun Contigs	97.88	100	*Neorhizobium galegae* strain NG_87_Ori (VZUL01)	Classification as *N. galegae* confirmed by phylogenomic analysis implemented in the Type Strain Genome Server (TYGS)
		96.75	100	*Neorhizobium galegae* bv. orientalis HAMBI 2605 (CCRM01)	NCBI notes: anomalous assembly
		98.05	65	*Rhizobiales* bacterium isolate R129_J (UCML01)	
		84.46	99	*Pararhizobium* sp. YC-54 (JAOYTH01)	
		76.36	100	*Rhizobiaceae* bacterium AM104-69 (JAQDQG01)	
	Standard nr/nt	76.43	99	*Shinella* sp. HZN7 plasmid pShin-06 (CP015742)	
		79.70	59	*Rhizobium daejeonense* strain KACC 13094 unnamed plasmid (CP086613)	

Supplementary Table 4. Growth characteristics of (1) *E. canadensis* sp. nov. T173^T, (2) *E. morelensis* Lc04^T, (3) *E. adhaerans* Casida A^T, (4) *E. sesbaniae* CCBAU 65729^T, (5) *E. medicae* A321^T, (6) *E. americanus* CFNEI156^T, (7) *E. fredii* USDA 205^T, and (8) *E. meliloti* ATCC 9930^T.

**Characteristic**	**1**	**2**	**3**	**4**	**5**	**6**	**7**	**8**
Growth on YEM agar medium*								
10 °C	±	±	±	-	-	-	-	-
37 °C	±	±	+	+	+	±	-	±
pH 5	+	+	+	+	±	±	±	±
pH 10	+	+	+	+	±	-	-	+
1% NaCl	+	+	+	+	+	±	±	+
2% NaCl	+	+	+	-	-	-	±	-
3% NaCl	-	-	-	-	-	-	-	-
Growth in LB broth*	+	+	+	+	±	±	±	+
Acid production †	+	ND	ND	+	+	+	+	+

* Positive, +; weak, ± ; negative, − ; not determined, ND. Values are based on three replicates.

† Acid production on YEM agar medium determined as described by Bromfield et al., (2010); pH values varied between pH 5 - 6.

Supplementary Table 5. Phenotypic characteristics (BIOLOG GEN III MicroPlates) of (1) *E. canadensis* sp. nov.T173^{T}, (2) *E. morelensis* Lc04^{T}, (3) *E. adhaerans* Casida A^{T}, (4) *E. sesbaniae* CCBAU 65729^{T}, (5) *E. medicae* A321^{T}, (6) *E. americanus* CFNEI156^{T}, (7) *E. fredii* USDA 205^{T}, and (8) *E. meliloti* ATCC 9930^{T}.

Characteristic	1	2	3	4	5	6	7	8	Characteristic	1	2	3	4	5	6	7	8
									C-source utilization								
Dextrin	-	+	+	+	-	-	-	+	Glycyl-L-Proline	-	+	±	±	-	-	-	-
D-Maltose	±	+	+	+	±	-	-	±	L-Alanine	-	-	±	-	-	-	-	-
D-Trehalose	±	+	+	+	-	-	-	+	L-Arginine	-	±	±	±	-	-	-	-
D-Cellobiose	±	+	+	+	±	-	±	+	L-Aspartic Acid	-	+	+	+	-	-	-	±
Gentiobiose	±	+	+	+	±	-	-	+	L-Glutamic Acid	±	±	±	+	-	-	-	±
Sucrose	±	+	+	+	-	-	-	+	L-Histidine	-	±	±	±	-	-	-	-
D-Turanose	-	-	-	-	±	-	-	-	L-Pyroglutamic Acid	-	-	-	-	-	-	-	-
Stachyose	-	-	-	-	-	-	-	-	L-Serine	-	±	±	±	-	-	-	-
D-Raffinose	±	±	+	±	-	-	-	-	Pectin	±	+	+	±	+	+	+	±
α-D-Lactose	±	±	+	±	-	-	-	+	D-Galacturonic Acid	-	-	-	-	-	-	-	±
D-Melibiose	±	±	±	-	-	-	-	±	L-Galactonic Acid Lactone	-	-	-	-	-	-	-	-
β-Methyl-DGlucoside	-	±	±	±	-	-	-	±	D-Gluconic Acid	±	-	-	-	-	-	-	-
D-Salicin	-	±	±	-	-	-	-	-	D-Glucuronic Acid	±	-	-	-	-	-	-	-
N-Acetyl-DGlucosamine	±	+	+	+	-	-	-	±	Glucuronamide	-	-	-	-	±	+	+	-
N-Acetyl-β-DMannosamine	±	-	±	-	-	-	-	±	Mucic Acid	-	-	-	-	-	-	-	-
N-Acetyl-DGalactosamine	-	±	±	±	-	-	-	-	Quinic Acid	±	±	-	±	-	-	-	-
N-Acetyl Neuraminic Acid	-	-	-	-	-	-	-	-	D-Saccharic Acid	-	-	-	-	-	-	-	-
α-D-Glucose	±	+	+	+	±	-	-	+	p-HydroxyPhenylacetic Acid	-	-	-	-	-	-	-	-
D-Mannose	±	+	+	+	-	-	-	+	Methyl Pyruvate	-	-	-	-	-	-	-	-
D-Fructose	±	+	+	±	±	-	-	+	D-Lactic Acid Methyl Ester	-	+	-	-	-	-	-	-
D-Galactose	-	±	±	±	±	-	-	±	L-Lactic Acid	-	+	±	±	-	-	-	-
3-Methyl Glucose	±	±	-	+	-	-	-	-	Citric Acid	-	-	-	-	-	-	-	-
D-Fucose	-	-	-	-	-	-	-	-	α-Keto-Glutaric Acid	-	-	-	-	-	-	-	-
L-Fucose	±	+	+	±	-	-	-	+	D-Malic Acid	±	+	±	+	-	-	-	±
L-Rhamnose	-	-	±	±	-	-	-	±	L-Malic Acid	+	+	+	+	-	-	-	+
Inosine	-	±	-	-	-	-	-	-	Bromo-Succinic Acid	-	-	±	-	-	-	-	-
D-Sorbitol	±	+	+	+	+	+	+	+	Tween 40	±	+	±	±	-	-	-	-
D-Mannitol	±	+	+	+	+	+	+	±	γ-Amino-Butryric Acid	-	-	-	-	-	-	-	-
D-Arabitol	+	+	+	+	+	+	+	+	α-HydroxyButyric Acid	-	-	-	-	-	-	-	-
myo-Inositol	+	+	+	+	±	+	+	+	β-Hydroxy-D,LButyric Acid	-	-	-	-	-	-	-	-
Glycerol	±	+	+	+	±	+	+	+	α-Keto-Butyric Acid	-	-	-	-	-	-	-	-
D-Glucose- 6-PO4	±	±	+	+	±	+	+	±	Acetoacetic Acid	+	+	±	±	-	-	-	+
D-Fructose- 6-PO4	±	-	±	-	±	+	+	-	Propionic Acid	±	-	-	+	-	-	-	+
D-Aspartic Acid	±	-	-	-	±	+	+	-	Acetic Acid	+	+	+	+	-	-	-	+
Gelatin	-	-	-	-	-	-	-	-	Formic Acid	±	-	-	-	-	-	-	-
									Chemical Sensitivity								
1% Sodium Lactate	-	+	+	-	-	±	-	-	Vancomycin	-	-	-	-	-	-	-	-
Fusidic Acid	-	-	-	-	±	+	+	-	Tetrazolium Violet	+	+	+	+	+	+	+	+
D-Serine	-	-	-	-	-	-	-	-	Tetrazolium Blue	+	+	+	+	+	+	+	+
Troleandomycin	±	+	+	+	-	+	+	-	Nalidixic Acid	-	-	±	+	±	-	-	-
Rifamycin SV	+	+	+	+	+	+	+	+	Lithium Chloride	-	-	-	-	-	-	-	-
Minocycline	-	-	-	-	-	+	+	-	Potassium Tellurite	-	±	-	+	+	±	-	-
Lincomycin	+	±	+	+	+	-	-	-	Aztreonam	+	+	-	-	+	-	-	-
Guanidine HCl	-	-	-	-	-	-	-	-	Sodium Butyrate	-	-	-	-	-	±	-	-
Niaproof 4	-	-	-	±	-	±	-	-	Sodium Bromate	-	-	-	-	-	±	-	-

Growth after 24 hours incubation at 30 ºC: +, positive; ±, weak; −, negative. Values are based on three replicates.

Supplementary Table 6. Examples of antibiotic resistance genes encoding enzymes that inactivate beta-lactam and amino-glycoside antibiotics detected in the genome of *E. canadensis* sp. nov. T173^{T}.

Gene (location)	Co-ordinates (bp)	Product	Function
bla (Chromosome - CP083370)	698,503-699,387	Class A beta-lactamase	Class A beta-lactamase<br>Classification: antibiotic inactivation enzyme<br>Antibiotic class: beta-lactam antibiotics
bla (pT173e - CP083371)	1,593,270-1,594,130	Subclass B3 beta-lactamase	Subclass B3 beta-lactamase<br>Classification: antibiotic inactivation enzyme<br>Antibiotic class: beta-lactam antibiotics
AAC(3)-II,III,IV,VI,VIII,IX,X (pT173c - CP083373)	24,137-24,955	Aminoglycoside N(3)-acetyltransferase	Aminoglycoside N(3)-acetyltransferase<br>Classification: antibiotic inactivation enzyme<br>Antibiotic class: aminoglycosides<br>Antibiotics: gentamicin C, tobramycin, gentamicin B, amikacin, kanamycin A, apramycin, neomycin.
AAC(6')-Ic,f,g,h,j,k,l,r-z (Chromosome - CP083370)	2,575,834-2,576,295	Aminoglycoside N(6')-acetyltransferase	Aminoglycoside N(6')-acetyltransferase<br>Classification: antibiotic inactivation enzyme<br>Antibiotic class: aminoglycosides<br>Antibiotics: tobramycin, kanamycin A, amikacin, dibekacin, sisomicin, gentamicin B, isepamicin, arbekacin, netilmicin, neomycin.
Unnamed (Chromosome - CP083370)	1,505,251-1,506,051	Aminoglycoside 6-phosphotransferase, (putative)	Aminoglycoside 6-phosphotransferase, (putative).<br>Classification: antibiotic inactivation enzyme<br>Antibiotic class: aminoglycosides
Unnamed (pT173e - CP083371)	3,349,865-3,350,656	Aminoglycoside 3'-hosphotransferase, (putative)	Aminoglycoside 3'-phosphotransferase, putative.<br>Classification: antibiotic inactivation enzyme<br>Antibiotic class: aminoglycosides
APH(3")-I (pT173e - CP083371)	155,136-155,939	Aminoglycoside 3"-phosphotransferase	Aminoglycoside 3"-phosphotransferase<br>Classification: antibiotic inactivation enzyme<br>Antibiotic class: aminoglycosides<br>Antibiotic: streptomycin

Data from the BV-BRC web-based platform (Olson et al., 2023)

Supplementary Table 7. Fatty acid profiles of (1) *E. canadensis* sp. nov. T173[T], (2) *E. morelensis* Lc04[T], (3) *E. adhaerans* Casida A[T], (4) *E. sesbaniae* CCBAU 65729[T], (5) *E. medicae* A321[T], (6) *E. americanus* CFNEI156[T], (7) *E. fredii* USDA 205[T], and (8) *E. meliloti* ATCC 9930[T].

Fatty Acid	1	2	3	4	5	6	7	8
13:1 at 12-13	0.07							
14:0	0.13	0.16			0.20			
15 :0 3OH	0.13			0.12				
16:0	7.70	6.40	4.15	4.09	12.40	8.01	7.65	7.61
16:0 3OH	1.56	2.06	2.59	1.73	1.37	0.90	0.81	0.91
17:0 cyclo	0.59	2.12		0.58	1.01	0.97	0.62	0.73
17:0 3OH	0.18			0.19				0.25
17:1 ω8c	0.16			0.14		0.36		0.14
17:0	0.62	0.27		0.37	0.32	0.63	0.72	0.65
18:0	4.34	1.82	2.28	1.72	2.81	2.53	5.60	2.09
18:0 3OH	2.17	1.98	4.24	3.17	3.19	3.50	4.17	4.30
18:1 w5c	0.12			0.22				0.20
18:1 ω7c 11-methyl	18.69	18.33	15.35	1.04	0.74	0.50	0.81	1.86
19:0	0.08							
19:0 cyclo ω8c	2.86	2.02	9.41	22.08	14.18	5.52	9.14	25.01
20:0 iso	0.20							
20:1 w7c	0.16			0.18				
20:2 w6,9c	0.31		0.85	0.81	0.64	0.48	0.77	1.46
Summed feature 1*	0.10							
Summed feature 2*	7.00	6.96	8.12	7.88	7.55	8.68	8.55	7.86
Summed feature 3*	2.44	2.19		0.45	1.03	2.23	0.91	0.51
Summed feature 5*	0.11							
Summed feature 7*	0.18			0.41				0.56
Summed feature 8*	50.07	55.69	53.01	54.82	54.56	65.70	60.24	45.86

* Summed Features are fatty acids that cannot be resolved reliably from another fatty acid using the chromatographic conditions chosen. The MIDI system groups these fatty acids together as one feature with a single percentage of the total. Summed feature 1, 15:1 iso H/13:0 3OH / 13:0 3OH/15:1 iso H; Summed feature 2, 12:0 aldehyde/?; Summed feature 3, 16:1 ω6c/16:1 ω7c; Summed feature 5, 18:0 ante/18:2 ω6,9c; Summed feature 7, 19:1w7c/19:1 w6c / 19:1 w6c/w7c/19cy; Summed feature 8, 18:1 ω6c/18:1 ω7c.

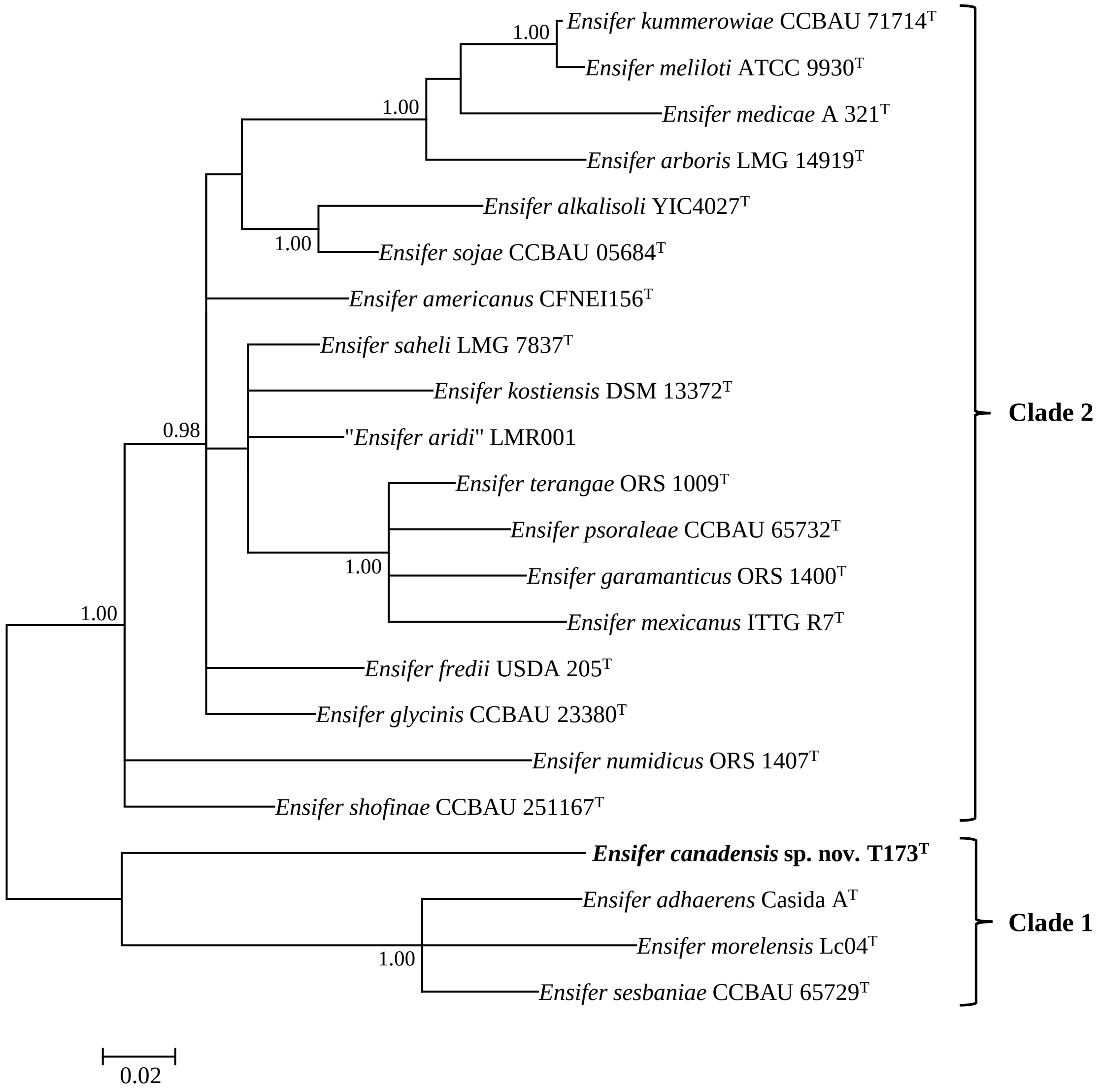

Supplementary Figure 1. Bayesian phylogenetic tree (GTR + G + I substitution model) of *glnII* gene sequences (615 bp) for *Ensifer canadensis* sp. nov. T173^{T} and reference taxa of the genus *Ensifer*. Only posterior probabilities ≥ 90% are shown. Bar, expected substitutions per site.

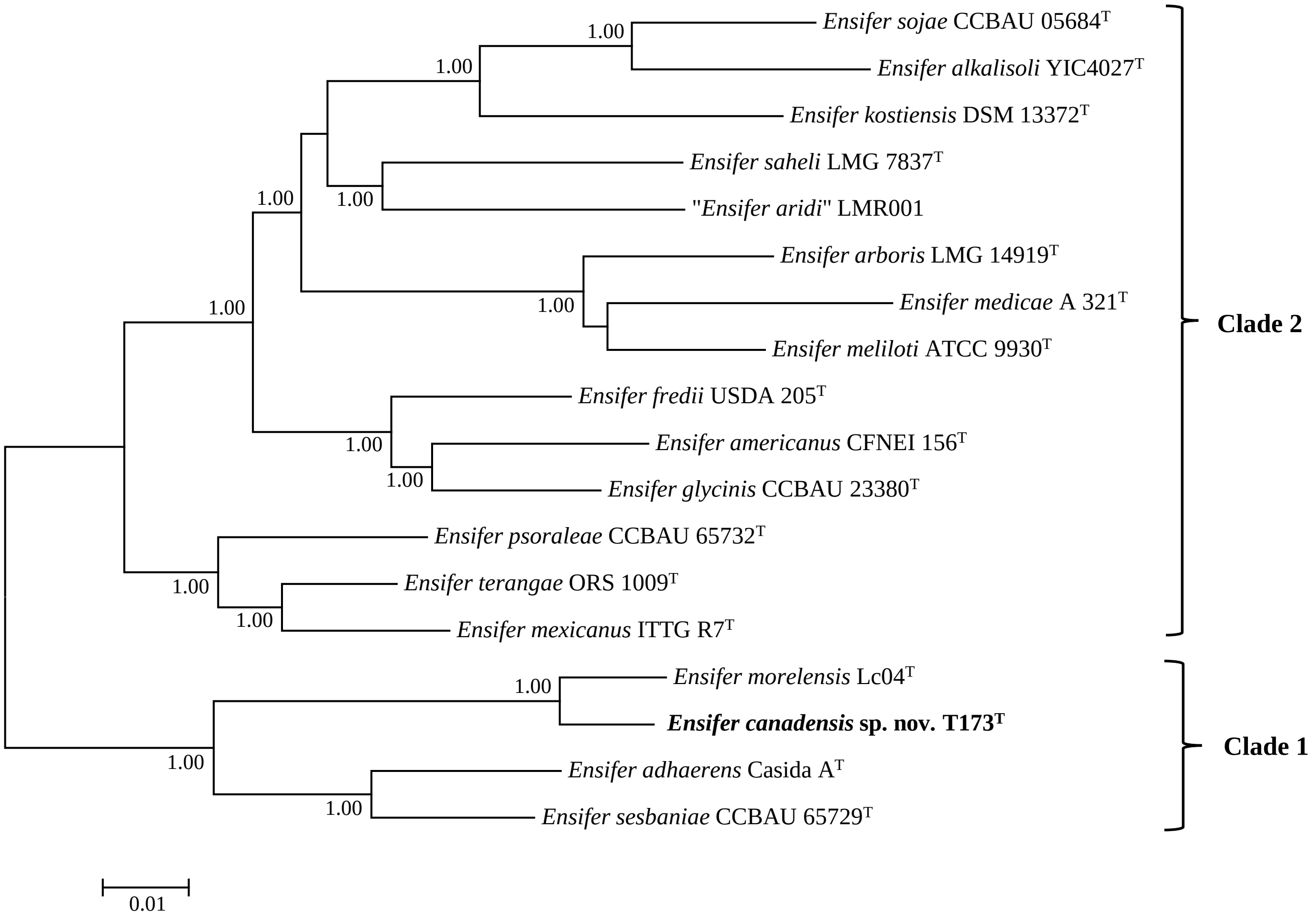

Supplementary Figure 2. Bayesian phylogenetic tree (GTR + G + I substitution model) inferred from 53 full-length concatenated ribosome protein subunit (*rps*) gene sequences of *Ensifer canadensis* sp. nov. T173^{T} and reference taxa of the genus *Ensifer*. Alignment length, 23067bp. Only posterior probabilities ≥ 90% are shown. Bar, expected substitutions per site.

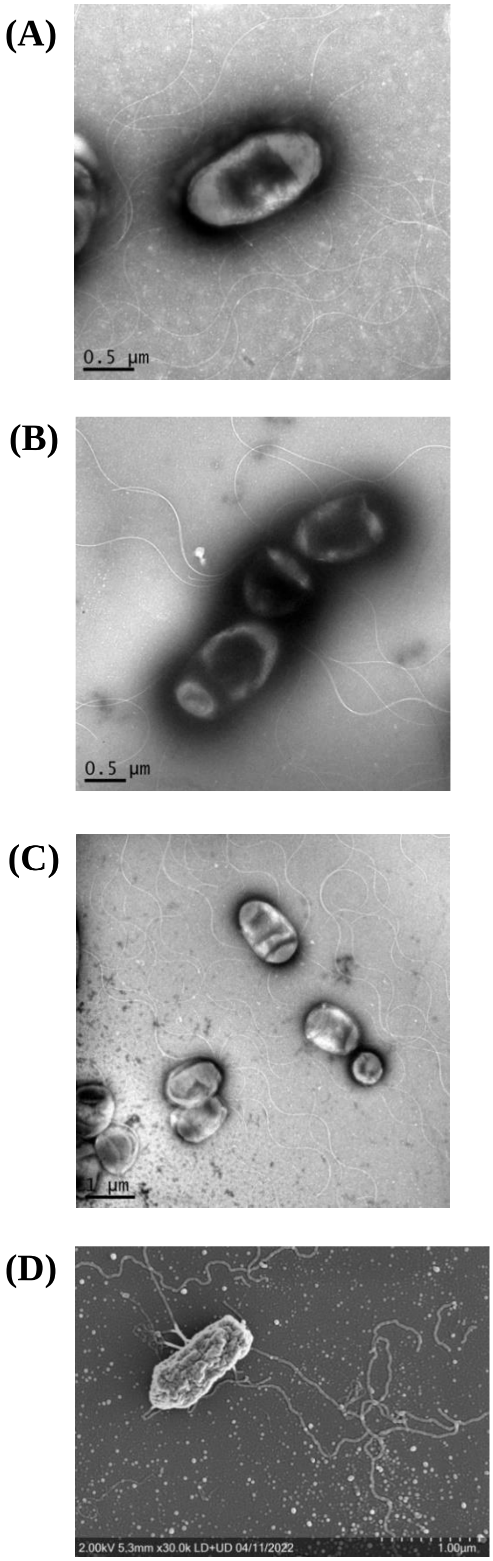

Supplementary Figure 4. Transmission (A, B and C) and scanning (D) electron microscope images showing cell morphological features of *Ensifer canadensis* sp. nov. strain T173^{T}.

Supplementary Figure 5. Growth curves (48 hrs at 30 °C) in LB broth medium of (1) *E.canadensis.* sp. nov.T173^T, (2) *E. adhaerans* Casida A^T, (3) *E. morelensis* Lc04^T, (4) *E. sesbaniae* CCBAU 65729^T, (5) *E. americanus* CFNEI156^T, (6) *E. fredii* USDA 205^T, (7) *E. medicae* A321^T, and (8) *E. meliloti* ATCC 9930^T.

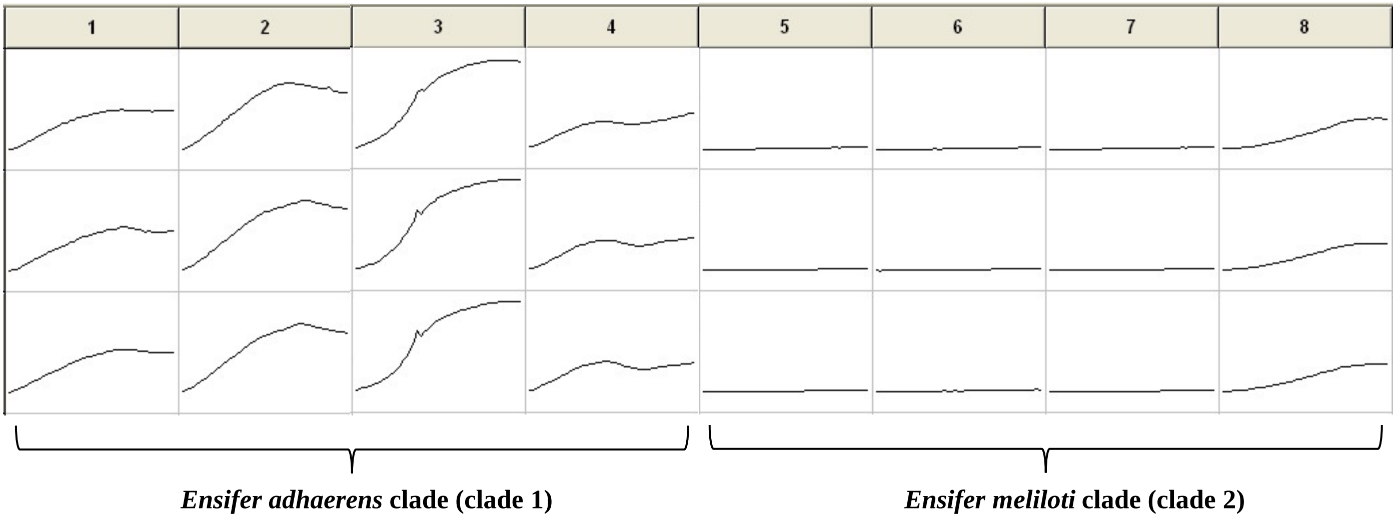

Vertical axis: bacterial growth based on OD (595 nm). Horizontal axis: hourly reads for 48 hours at 30 °C (three replicates of each bacterial strain).
